# Commentary on the paper ‘Modelling determinants, impact and space–time risk of age-specific mortality in rural South Africa: integrating methods to enhance policy relevance’

**DOI:** 10.3402/gha.v6i0.19882

**Published:** 2013-01-25

**Authors:** Elisabeth Dowling Root

**Affiliations:** Department of Geography and Institute of Behavioral Science, University of Colorado at Boulder, Boulder, CO, USA

Although the study of small-area variations in health has a long history, only recently have spatial statistical methods been used to examine spatial patterns of morbidity and mortality. As far back as the 1600s, public health researchers recognized that people living in different areas had differing health outcomes (1, 2). However, the availability of desktop mapping and increasingly powerful statistical software has led to a growing interest in statistical methods for the analysis of spatially referenced public health data. Spatial statistical methods offer a means for us to use locational information, such as latitude/longitude, municipality, or postal code, to detect and quantify patterns in public health data and to investigate the degree of association between potential risk factors and disease. The identification and quantification of patterns of disease occurrence provide the first steps toward increased understanding and possible control of disease (3).

However, diseases also have a context, and it is important to determine not only the patterns that exist but also the underlying risk factors that contribute to those patterns. Observable differences in health outcomes between places may be due to differences in the kinds of people who live in these places (composition) or differences in the physical or social environment (contextual) (4–6). Spatial patterns can arise because people with similar individual-level risk factors tend to live near each other, resulting in larger spatially observable patterns of disease. However, if a disease is impacted by aspects of the environment, such as social and material deprivation, access to health services, or environmental contamination, spatial patterns may be due to the context within which people live. Spatial statistical methods, such as those employed by Dr. Benn Sartorius in this paper, are one way by which public health researchers can tease out the differential effects of compositional and contextual factors on mortality.

Spatial analysis necessitates the availability of geographically referenced data. The collection of geographic data is understandably not a priority for a public health surveillance system in most developing countries. Most large-scale geographic analysis of disease in developing countries is done at a relatively coarse geographic resolution, using provinces, municipalities, or districts as the level of analysis. Small-scale or local-level analysis is typically only conducted if geographic data are collected (e.g. via GPS units) as part of a directed research project. Most Health and Demographic Surveillance Systems (HDSS), including Agincourt, now collect the location of all households within the study site and store this information in a geographic information system (GIS). This study takes full advantage of the Agincourt GIS to conduct a sophisticated spatial analysis of age-specific mortality. Not only does the study examine the spatial patterns of age-specific mortality, but Dr. Sartorius also links individual-, household-, and village-level risk factors to these mortality rates. Such information is invaluable for public health planning and program implementation.

Kriging is a geostatistical method for creating a smoothed surface out of point data and is often employed in public health to create ‘risk surfaces’ or disease maps. Kriging typically requires very large datasets in order to create accurate estimates, which would be problematic for a small area such as Agincourt. This paper employs a more advanced statistical technique, Bayesian kriging, which provides more accurate estimates for small datasets. The maps of all-cause mortality included in the paper point to clear geographic differences in relative risk across the study site. The Agincourt study site is not large (approximately 400 km^2^) and health services are available; hence, it is remarkable that even in a study area of this size obvious clusters of mortality can be seen. In addition, the spatial patterns are different for infants, children, and adults. The highest areas of risk for infants occur in the northeast, a pattern not seen with adults.

The series of all-cause mortality maps presented in this paper (Figure 4) have implications for public health intervention and program implementation. In countries with finite public health resources, highlighting areas of particularly high disease or mortality risk can reveal areas where resources can be utilized most effectively. Maps of specific health outcomes (such as the HIV maps which Dr. Sartorius mentions but is unable to publish due to possible stigmatizing effects) are invaluable for planning and implementing specific health interventions, such as HIV education or ART programs. In addition, examining maps over time can reveal the impact of interventions. For example, the disappearance of HIV/TB clusters in the years subsequent to the rollout of HIV/TB and antiretroviral programs may suggest that the program is having a positive effect.

While observing the patterns of mortality is extremely important, this study takes the spatial exploration of mortality one step further by conducting a statistical analysis of the risk factors that contribute to age-specific mortality. In this, Dr. Sartorius adds context to his study and attempts to tease apart the compositional and contextual factors that may be responsible for the spatial patterns of mortality that he observes. Most notably, he finds that poor household socio-economic status and increased distance to the nearest health facility leads to higher mortality risk among adults.

While the results may not look terribly different from any other multivariate regression analysis, the underlying models used to derive the estimates are quite unique. They are true spatial models, in that they not only incorporate geographic variables such as distance to health facility but also adjust for the statistical biases caused when data are spatially correlated. Every researcher learns in their introductory multivariate regression course that all observations must be independent and identically distributed (*iid*) in order for estimates to be accurate. Spatially referenced health data almost always violate this assumption because people in nearby locations are often exposed to the same risk factors and exhibit similar disease outcomes. This is often termed ‘spatial autocorrelation’ and can cause bias in the standard error estimates of general linear models (7).

In this paper, Dr. Sartorius employs a class of geostatistical models that utilize a spatial random effect and a covariance structure that incorporates the distance between villages in Agincourt (again, derived from the GIS). In this way, we can have more confidence in the estimates in his results because he has controlled for the spatial correlation between mortality events in the dataset. Often, an estimate of the spatial random effect is also included in the results, which is not done in this paper, so that we can begin to understand how much variation in mortality exists between different villages and if these differences are statistically significant. The only drawback of the spatial methods this paper uses is that they are complex, and sometimes difficult to understand, so a strong grasp of Bayesian statistics is needed to execute and interpret model results. In addition, geographic data must be available, so that the distance between individuals or villages can be incorporated into the model.

This paper finds that all-cause mortality has a clear spatial pattern in Agincourt, which differs by age group. In addition, it confirms that a complex set of interactions between village-, household-, and individual-level risk factors drive these spatial patterns. It is not only one of the most comprehensive statistical analyses of mortality in Agincourt over time but perhaps more importantly, it demonstrates the usefulness of spatial statistical analysis in the field of public health. Results should be used to spatially target age-specific intervention programs and provide health services.
